# Expression of the myeloid inhibitory receptor CLEC12A correlates with disease activity and cytokines in early rheumatoid arthritis

**DOI:** 10.1038/s41598-021-90631-7

**Published:** 2021-05-27

**Authors:** Myriam Vaillancourt, Philippe Desaulniers, Guillaume Paré, Nathalie Pagé, Asmaa Lachhab, Anthony Kerever, Anne-Sophie Julien, Nathalie Amiable, Martin Pelletier, Philippe A. Tessier, Louis Bessette, Laëtitia Michou, Paul R. Fortin, Maria J. Fernandes

**Affiliations:** 1 Division of Infectious Diseases and Immunology, CHU de Québec-Université Laval Research Center, Québec City, QC Canada; 2 Department of Microbiology and Immunology, Faculty of Medicine, Université Laval, Québec City, QC Canada; 3 Division of Rheumatology, Department of Medicine, CHU de Québec-Université Laval Research Center, Québec City, QC Canada; 4 Department of Mathematics and Statistics, Université Laval, Québec City, QC Canada; 5 Division of Endocrinology and Nephrology, CHU de Québec-Université Laval Research Center, Québec City, QC Canada; 6 Centre de Recherche du CHU de Québec-Université Laval, 2705 boulevard Laurier, Room T1-49, Québec, QC G1V 4G2 Canada

**Keywords:** Biological techniques, Cell biology, Immunology, Diseases, Pathogenesis, Rheumatology

## Abstract

The myeloid inhibitory receptor CLEC12A negatively regulates inflammation. Reduced CLEC12A expression enhances inflammation in CLEC12A knock-out mice with collagen antibody-induced arthritis. Moreover, CLEC12A internalisation augments human neutrophil activation. We thus postulated that CLEC12A expression on circulating myeloid cells of rheumatoid arthritis patients is associated with disease manifestations. Cell-surface, CLEC12A receptor expression was determined on circulating neutrophils and monocytes of eRA patients and of healthy donors. Generalized estimating equations model, Student’s *t*-test and Spearman’s correlations were performed to compare CLEC12A expression between groups and test its association with disease activity and clinical parameters. Plasma cytokines were measured by multiplex immunoassay. Patients with reduced neutrophil or monocyte CLEC12A expression at baseline and at 3 months have an increased simple disease activity index. Low baseline CLEC12A expression also correlates with a higher SDAI at 6 months. In contrast, positive correlations were observed between baseline CLEC12A expression and several cytokines. Moreover, neutrophil and monocyte CLEC12A expression is significantly higher in early rheumatoid arthritis patients at baseline than healthy controls. Circulating neutrophil and monocyte CLEC12A expression correlates with disease activity at baseline and is predictive of SDAI at later stages of the disease indicative of a regulatory role for CLEC12A in RA.

## Introduction

Rheumatoid arthritis (RA) is one of the most common, systemic autoimmune diseases with a worldwide prevalence of 1%^1^. This chronic inflammatory disease develops as a result of complex interactions between environmental and several genetic factors^1^. Smoking and HLA-DR polymorphisms^2^ are among the main risk factors that together promote the development of autoantibodies of which anti-citrulline antibodies are the most specific for RA. Autoantibodies drive joint inflammation by activating resident immune cells and synoviocytes that in turn, promote neutrophil and monocyte recruitment and activation by releasing cytokines^2^.

The most abundant leukocyte in RA synovial fluid, the neutrophil, contributes to all stages of the pathogenesis of RA^3–5^. At mucosal sites such as the lung, neutrophils facilitate the initial loss of tolerance in genetically susceptible individuals by contributing to the development of autoantigens. In the inflamed joint, neutrophils contribute to the generation of autoantibodies by activating the antigen-presenting function of synoviocytes by releasing neutrophil extracellular traps^5^. Neutrophils also cause joint damage in RA by releasing serine proteases and matrix metalloproteinases (e.g. MMP-8 and MMP-9) and by generating toxic reactive oxygen species (ROS) via NADPH oxidase^5^. Activated fibroblasts and neutrophils promote neutrophil and monocyte recruitment from the circulation by releasing cytokines (e.g. IL-8). Recruited monocytes contribute to RA through their antigen presentation properties, pro-inflammatory cytokine production and their differentiation into bone-resorbing cells^6^. The key role of neutrophils and monocytes in RA underscores the importance of functional counter-regulatory mechanisms to control the activation of these myeloid cells during an immune response to avoid developing immune-mediated diseases.

One of RA’s fundamental underlying causes is a dysregulation of immune-activating and immune-inhibitory pathways^7^. CLEC12A is one of the few myeloid inhibitory receptors known to contribute to the pathogenesis of RA^8–13^. It possesses an extracellular C-type lectin domain with an intracellular immunoreceptor tyrosine-based inhibitory motif through which it recruits phosphatases to downregulate activating pathways^12^. The loss of CLEC12A expression in CLEC12A knock-out (KO) mice leads to increased joint inflammation, neutrophil activation, and impaired joint injury resolution caused by collagen-induced arthritis compared to wild-type mice^14^. Similarly, antibody-induced internalisation of cell-surface CLEC12A in wild-type mice exacerbates the collagen-induced arthritis phenotype^14^. Neutrophil recruitment in CLEC12A KO mice is also significantly increased during monosodium urate crystal-induced inflammation^15^. Moreover, antibody-induced internalisation of cell-surface CLEC12A in human neutrophils enhances neutrophil responses to monosodium urate crystals^16^. CLEC12A thus regulates neutrophil responses, in part, by diminishing its cell-surface expression.

While these observations support an essential regulatory role for CLEC12A in determining disease severity in RA by modifying its expression on neutrophils and potentially other myeloid cells, CLEC12A expression on circulating early RA (eRA) leukocytes remains unexplored. The purpose of this study was thus to determine whether levels of CLEC12A on circulating neutrophils and monocytes of eRA patients are associated with disease activity, serum cytokine levels and/or other clinical parameters.

## Results

### Patient characteristics

Baseline characteristics of the 17 eRA patients of our discovery cohort are shown in Table 1. Patients were diagnosed as per 2010 ACR/EULAR criteria**.** Thirty percent of patients were negative for both rheumatoid factor (RF) and anti-CCP antibodies. One patient was negative for anti-CCP and positive for RF. Most patients were disease-modifying anti-rheumatic drugs (DMARDs) naïve. One patient was on methotrexate and three patients received prednisone before the baseline visit. After diagnosis at baseline, the majority of eRA patients (14/17) were treated with methotrexate (aiming for a dose of 20 mg/week) and some with prednisone as well (6/17). Response to treatment was evaluated during the 6-month follow-up visits. Remission was defined as a simple disease activity index (SDAI) score ≤ 3.3. For comparative purposes, healthy donors were also recruited (Table 2).

**Table 1:** Early RA patient cohort: characteristics at baseline.

	Total (n = 17)
Age, years, mean (SD)	61.06 (11.65)
Female, n (%)	10 (58.8)
Caucasian, n (%)	16 (94)
BMI, mean (SD)	26.72 (4.6)
Smoker, n (%)	0 (0)
Ex-smoker, n (%)	12 (80)
Symptoms duration, years (SD)	0.51 (0.22)
ESR, mm/h, mean (SD)	13.71 (10.80)
CRP, mg/L, mean (SD)	8.95 (12.52)
RF, KUI/L, mean (SD)	90.24 (97.16)
RF +, n (%)	12 (71)
Anti-CCP, u/ml, mean (SD)	153.41 (118.17)
Anti-CCP +, n (%)	11 (65)
SJC28, mean (SD)	10.47 (6.51)
SDAI, mean (SD)	34.48 (14.73)
Prednisone, n (%)	3 (18)
Methotrexate, n (%)	1 (6)

*Anti-CCP* anti-cyclic citrullinated peptide, *BMI* body mass index, *CRP* C-reactive protein, *ESR* erythrocyte sedimentation rate, *RF* rheumatoid factor, *SDAI* simple disease activity index, *SJC28* swollen 28-joint count, % percentage after excluding missing values.

**Table 2:** Healthy donors’ characteristics.

	Total (n = 16)
Age, years, mean (SD)	52.56 (7.86)
Female, n (%)	11 (68.8)
BMI, mean (SD)	26.44 (5.28)
Smoker, n (%)	0 (0)
Ex-smoker, n (%)	1(6.25)

*BMI* body mass index, % percentage after excluding missing values.

### Neutrophil CLEC12A expression correlates with SDAI

**Figure 1:**
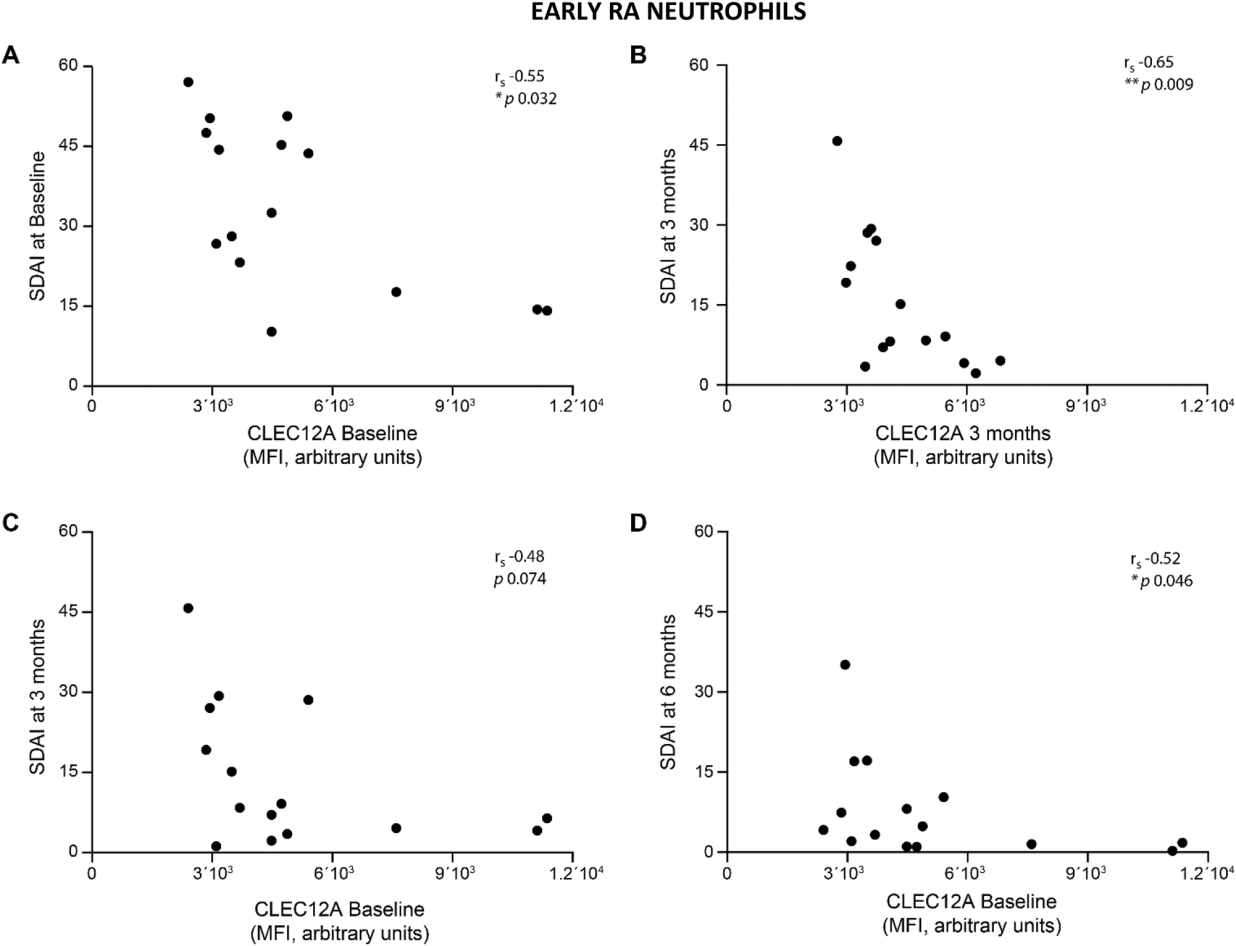
Neutrophil CLEC12A expression in eRA patients correlates with SDAI. Cell-surface, CLEC12A expression was determined by flow cytometry on circulating neutrophils of eRA patients. Cross-sectional correlations between neutrophil CLEC12A expression and SDAI score in eRA patients at **(A)** baseline, and **(B)** three months. Correlations between baseline, neutrophil CLEC12A expression and SDAI at **(C)** three and **(D)** six months. Statistical analysis: Spearman’s rank correlation coefficient and *p* value are shown in each panel. Symbols represent individual patients.

Since reduced CLEC12A expression in CLEC12A KO mice with collagen antibody-induced arthritis enhances neutrophil activation and inflammation^14^, we determined whether neutrophil CLEC12A expression in eRA patients correlates with clinical parameters. CLEC12A expression of peripheral leukocytes of eRA patients was determined by flow cytometry after staining whole blood with an anti-CLEC12A antibody and a leukocyte-specific antibody panel (Additional file 1). Comparison of CLEC12A expression with disease activity revealed that patients with lower levels of baseline CLEC12A on circulating neutrophils have a significantly higher SDAI score (Fig. 1A). Similarly, at 3-months follow-up, reduced neutrophil CLEC12A expression also correlates with a significantly higher SDAI score (Fig. 1B). In addition, a lower level of baseline CLEC12A expression on circulating neutrophils also correlates with a higher SDAI score at 6 months but not at 3 months (Fig. 1C,D). CLEC12A expression thus correlates with SDAI at baseline and at defined time-points in response to treatment.

### Monocyte CLEC12A expression correlates with SDAI

**Figure 2:**
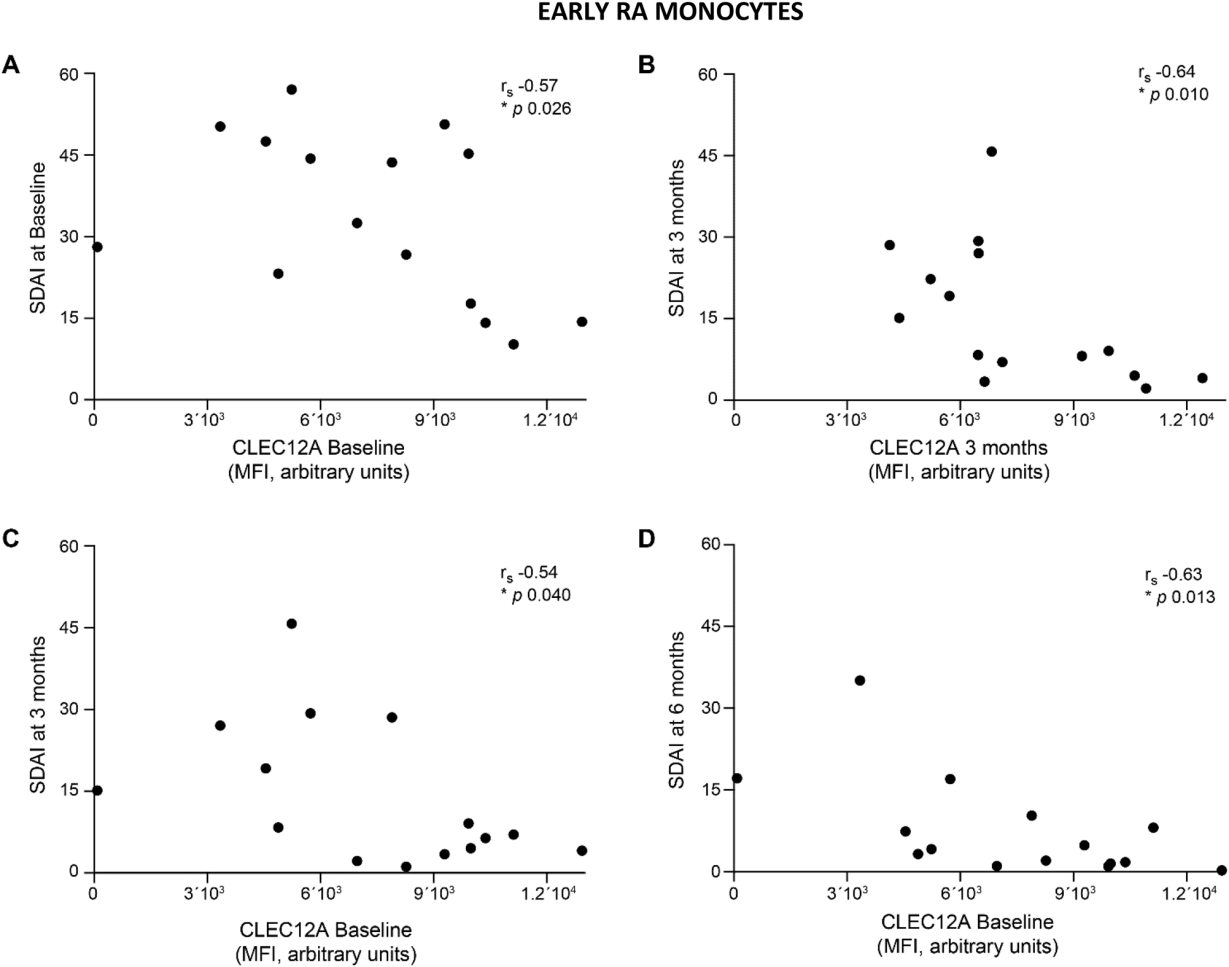
Monocyte CLEC12A expression in eRA patients correlates with SDAI. Cell-surface, CLEC12A expression was determined by flow cytometry on peripheral monocytes. Cross-sectional correlations between monocyte CLEC12A expression in eRA patients and SDAI score at **(A)** baseline, and **(B)** three months. Correlations between baseline, monocyte CLEC12A expression and SDAI at **(C)** three months and **(D)** six months. Statistical analysis: Spearman’s rank correlation coefficient and *p* value are shown in each panel. Symbols represent individual patients.

Since the enhanced disease manifestations in CLEC12A KO mice with collagen antibody-induced arthritis were due to a lack of CLEC12A in the myeloid compartment^14^, we also analyzed CLEC12A expression in circulating monocytes of eRA patients. Similar to neutrophils, patients with lower monocyte CLEC12A expression on circulating monocytes at baseline or at 3 months follow-up have a higher SDAI score (Fig. 2A,B). Moreover, baseline monocyte CLEC12A expression negatively correlates with the SDAI score at 3 and 6 months (Fig. 2C,D). The negative correlation between CLEC12A expression and SDAI is thus observed in at least two circulating myeloid populations, neutrophils and monocytes.

### Neutrophil and monocyte CLEC12A expression correlates with cytokine levels

Since reduced CLEC12A expression increases cytokine expression in neutrophils and dendritic cells^9,16^, we next explored the possibility that CLEC12A expression on circulating eRA neutrophils and monocytes correlates with levels of cytokines pertinent to the pathology of eRA^17^. The great majority of cytokines detected in our eRA cohort have been reported with some consistency in other eRA cohorts^18^. Of the cytokines detectable in the majority of eRA patients and/or healthy controls at baseline, eotaxin/CCL11 correlated positively with neutrophil and monocyte CLEC12A expression (r_s_ = 0.71; *p* = 0.003 and r_s_ = 0.55; *p* = 0.034 respectively) (Table 3 and Additional file 2). At baseline, monocyte MIP-1β/CCL4 and IL-1-RA levels also correlated positively (r_s_ = 0.58; *p* = 0.024 and r_s_ = 0.53; *p* = 0.043 respectively) with CLEC12A expression while RANTES/CCL5 (r_s_ = -0.67; *p* = 0.007) expression correlated negatively with CLEC12A. In contrast, baseline IL-18, IL-1β, IP-10/CXCL10, MCP-1/CCL2, MIP-1α/CCL3, or SDF-1α/CXCL12 levels did not correlate with the expression of CLEC12A *(data not shown)*. Likewise, no correlation was observed in healthy donors between cytokine levels and CLEC12A expression (n = 10, *data not shown*).

**Table 3:** Correlations between neutrophil and monocyte CLEC12A expression and cytokine levels at baseline in early RA patients.

	Neutrophil CLEC12A		Monocyte CLEC12A	
	r_s_	*P*	r_s_	*P*
Eotaxin/CCL11	0.71	0.003	0.55	0.034
MIP-1β/CCL4	0.34	0.211	0.58	0.024
IL-1-RA	0.44	0.101	0.53	0.043
RANTES/CCL5	− 0.46	0.084	− 0.67	0.007

r_s_ Spearman’s correlation coefficient, *P p* value, cytokines were analyzed as continuous variables, *n* = 15.

Cytokines detected in at least two eRA patients were analyzed as dichotomic values. These included IL-1α, IL-2, IL-6, IL-7, IL-8, IL-10, IL-13, IL-15, IL-17A/CTLA-8, IL-21, IL-22, IL-27, Gro-α/CXCL1, IFN-α, IFN-γ and TNF-alpha. No associations were detected between baseline neutrophil and monocyte CLEC12A expression and baseline levels of these cytokines (*data not shown).* Cytokines not detected in any samples included IL-4, IL-5, IL-9, IL-12p70, IL-23, IL-31, TNF-β and GM-CSF. Altogether, these findings indicate that there exists a potential link between CLEC12A expression and the production of a select number of cytokines involved in eRA.

### Downregulation of neutrophil and monocyte CLEC12A expression with eRA treatment

**Figure 3:**
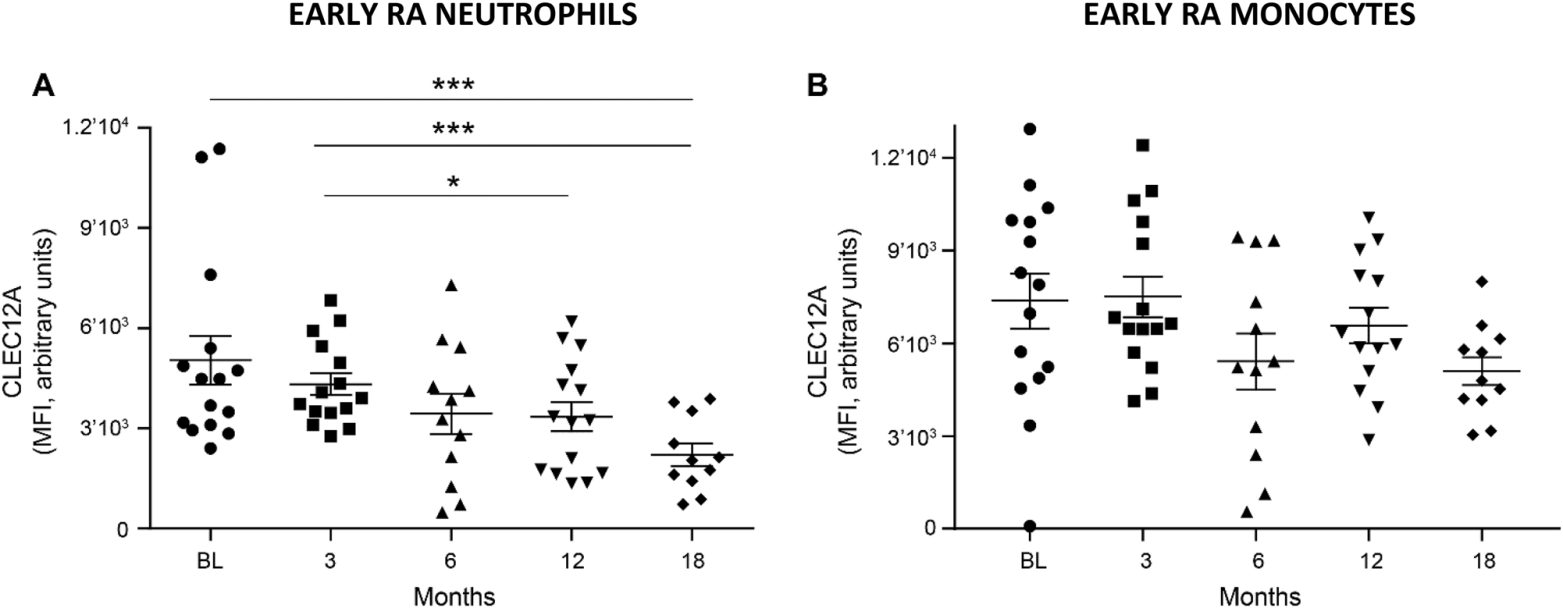
Neutrophil and monocyte CLEC12A expression diminishes with treatment in eRA patients. Cell-surface, CLEC12A expression was determined by flow cytometry on peripheral neutrophils and monocytes and analyzed with GEE models and Bonferroni correction. **(A)** Analysis of neutrophil CLEC12A expression revealed significant differences between groups (*p* = 0.017). Pairwise significant difference *p* values are 0.0007 for baseline (BL) and 18 months, 0.018 for 3 and 12 months and < 0.0001 for 3 and 18 months. **(B)** Analysis of monocyte CLEC12A expression did not reveal significant differences between groups (*p* = 0.081). Symbols represent individual patients at baseline (BL) and after 3, 6, 12 and 18 months of treatment. Data show the mean ± SEM. *** < 0.001 ** < 0.01 * < 0.05.

We then tested the hypothesis that CLEC12A expression changes during disease progression as clinical outcomes that could be influenced by CLEC12A, such as joint inflammation, diminish with treatment. Cell-surface CLEC12A expression on circulating neutrophils of eRA patients diminished with treatment and reached significance at 18 months compared to baseline (Fig. 3A). CLEC12A expression on eRA neutrophils reached healthy donor levels at 18 months (Additional file 3). A similar downregulation of CLEC12A expression was observed on eRA monocytes but did not reach significance between any two-time points, most likely due to the more heterogeneous expression of CLEC12A on circulating eRA monocytes compared to neutrophils (Fig. 3B). Together, our observations indicate a concomitant decrease in CLEC12A expression and disease activity with treatment.

### Neutrophil and monocyte CLEC12A expression differs between eRA patients and healthy donors

**Figure 4:**
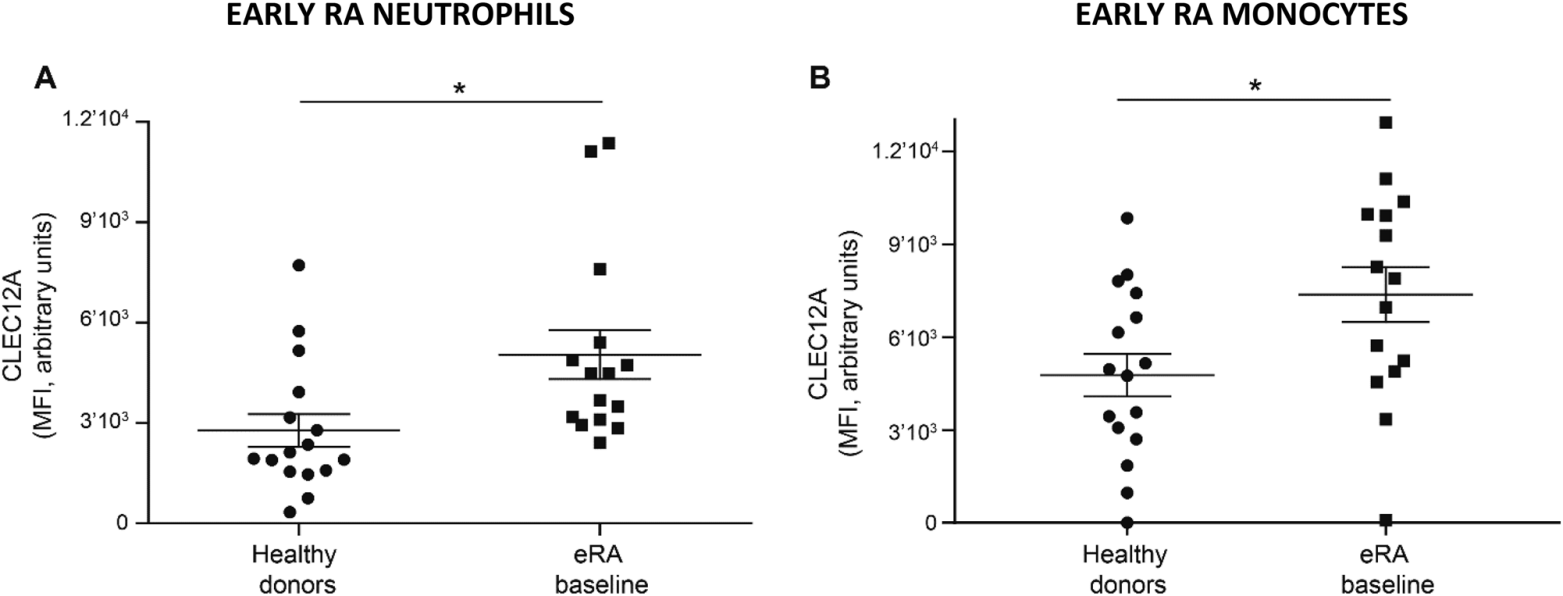
CLEC12A is differentially expressed on eRA neutrophils and monocytes compared to healthy donors. CLEC12A expression was measured by flow cytometry on peripheral neutrophils and monocytes of eRA patients and healthy donors. **(A)** Comparison of neutrophil CLEC12A expression of healthy donors and eRA patients at baseline (Student’s *t*-test, *p* = 0.014). **(B)** Comparison of monocyte CLEC12A expression of healthy donors and eRA patients at baseline (Student’s *t* test, *p* = 0.026). Symbols represent individual subjects. Data show the mean ± SEM. *** < 0.001 ** < 0.01 * < 0.05.

CLEC12A expression was then compared between eRA patient and healthy donor neutrophils and monocytes. As shown in Fig. 4, baseline CLEC12A expression is significantly higher on circulating neutrophils and monocytes of eRA patients than healthy donors. Since CLEC12A is not only expressed on the surface of neutrophils but also stored in cytoplasmic granules^19^, Western blot analysis was performed to determine whether the increase in cell-surface CLEC12A is due to its de novo synthesis or its translocation from gelatinase granules to the cell surface. Similar to the flow cytometry data, we observed an increased amount of endogenous CLEC12A in neutrophil lysates of eRA patients at baseline compared to healthy donors (Additional file 4). Together, these observations indicate that CLEC12A expression is upregulated in circulating eRA neutrophils and monocytes.

## Discussion

Inhibitory receptors on neutrophils are exquisite sensors of changes in the environment as their expression changes rapidly upon myeloid cell activation. We identified the inhibitory receptor CLEC12A as a systemic indicator of disease activity in eRA patients. Reduced CLEC12A expression on circulating neutrophils and monocytes correlates with a higher SDAI score. Moreover, we also provide evidence that CLEC12A expression correlates with cytokine levels in circulation. Since lower CLEC12A plasma membrane expression enhances neutrophil responses, these data suggest that CLEC12A contributes to disease activity (SDAI) by regulating the activation of neutrophils and monocytes.

While lower neutrophil and monocyte CLEC12A expression at baseline or 3 months correlates with a higher SDAI score, it did not correlate with anti-CCP or RF positivity *(data not shown)*. These observations suggest that CLEC12A may be an alternative disease activity indicator that is independent of serological reactivity. While no correlations between CLEC12A expression and baseline levels of ESR or CRP levels were identified, it is of note that the levels of these inflammation markers were within the normal range for all patients *(data not shown)*. Further experimentation in a cohort of eRA patients with higher levels of these markers will reveal whether CLEC12A on circulating leukocytes is a more specific marker of disease activity than ESR and CRP that are known to not always accurately reflect joint inflammation^20^.

The correlation between CLEC12A expression and circulating cytokine levels in eRA patients provides insight into potential molecular mechanisms underlying the association between CLEC12A and SDAI. The positive correlation between CLEC12A and eotaxin/CCL11 levels is relevant to the pathogenesis of eRA as low eotaxin/CCL11 levels were previously associated with increased radiographic progression in RA^21^. CLEC12A expression also correlates with IL-1RA, a cytokine that can tip the balance from pro-inflammatory to inhibitory signalling and potentially retard RA’s progression. IL-1RA counteracts IL-1 responses by binding the IL-1 receptor without initiating any signalling. IL-1 contributes to RA, in part, by activating endothelial cells of the vasculature and driving bone resorption^17^. Since systemic cytokines reflect the sum of cytokines produced by peripheral leukocytes and cytokines transiting from distant tissue sites, it remains to be determined whether CLEC12A directly modulates the production of cytokines by blood myeloid cells. Our data thus indicates that CLEC12A expression correlates with a subset of cytokines underscoring the specificity of the association between cytokines and this inhibitory receptor.

Our observation that CLEC12A expression on circulating monocytes and neutrophils of eRA patients is significantly higher than healthy donors may seem counterintuitive. These findings can be explained by the compartment in which CLE12A expression was measured. Neutrophils are wired to become fully activated once they arrive at the site of inflammation. Their activation in the circulation is tightly controlled by counter-regulatory mechanisms such as inhibitory receptors as it would be harmful to the host if neutrophils were to release their defences (e.g. reactive oxygen species and proteases) in the blood^22^. It thus follows that neutrophils whose CLEC12A expression increases in circulation to counteract the subclinical inflammatory environment of eRA have a functional CLEC12A pathway. We interpret these results to indicate that those patients whose neutrophils do not respond to the increase in pro-inflammatory stimuli in circulation by raising their CLEC12A expression, are neutrophils whose counter-regulatory mechanisms are functionally deficient and that consequently respond more robustly at the site of inflammation to activation signals increasing the SDAI. A lower level of CLEC12A expression on circulating neutrophils and monocytes thus correlates with a higher SDAI.

The strength of our study on our eRA discovery cohort is twofold. First, our findings align with previous observations reported in two models of arthritis in CLE12A KO mice^14,15^ and in in vitro assays with human neutrophils^16^. In all these studies, a decrease in CLEC12A leads to an enhancement of neutrophil activation as well as inflammation in vitro and in vivo, respectively. Secondly, low baseline CLEC12A expression levels correlate with a higher SDAI at 6 months. The predictive potential of CLEC12A for disease activity during the progression of eRA is of clinical interest and thus merits confirmation in a validation cohort.

Our findings not only underscore the role of CLEC12A in RA but also of myeloid inhibitory receptors, a less well characterized facet of this disease compared to lymphocyte inhibitory receptors. To our knowledge, the other C-type lectin, myeloid inhibitory receptor that is associated with RA is DCIR (dendritic cell immunoreceptor)^23^. In contrast to CLEC12A, DCIR is not differentially expressed on granulocytes and monocytes of RA patients. A likely explanation for these opposing observations is that the DCIR study was performed in a cohort of established RA patients. It remains to be determined whether correlations of CLEC12A expression with clinical parameters made in our eRA cohort will also be observed in established RA.

In summary, we identified CLEC12A expression as a potential, systemic indicator of disease activity in eRA patients. The correlations between CLEC12A expression and cytokine levels strongly suggest that CLEC12A modulates the immune response that drives the early stages of this autoimmune disease. Since reduced CLEC12A plasma membrane expression enhances neutrophil responses, these data suggest that pharmacologic manipulation of CLEC12A plasma membrane expression on neutrophils is a potential therapeutic strategy to treat eRA and possibly other chronic inflammatory diseases. These observations indicate that a higher expression of CLEC12A increases the threshold of activation of peripheral neutrophils and monocytes resulting in lower disease activity.

## Methods

### Patients and healthy donors

Early RA patients were defined as patients with a disease duration of > 6 weeks but < 12 months from the start of symptoms as in the Canadian Early Arthritis Cohort^24^ and recruited by SARD-BDB (Systemic Autoimmune Rheumatic Disease biobank and database repository of the CHU de Québec-Université Laval). Healthy donors were also recruited (n = 16, mean age of 53 ± 8 years, 69% female) with frequency matching regarding age and sex (Table 2). A sample size calculation was not performed for this discovery cohort.

### SARD-BDB protocol

Clinical data, blood and plasma samples were obtained from eRA patients at the first visit (baseline) and at the 3, 6, 12 and 18 month follow-up visit. A one-time control sample was obtained from healthy donors. Samples were harvested according to our established Standard Operating Procedures. Briefly, all clinical samples were processed within 2 h of collection. Blood was drawn in several 7 ml Vacutainer Buff sodium citrate (0.109 M, 3.2%) tubes that were gently inverted 4 times prior to being processed. For plasma preparation, two of these tubes were centrifuged at 282×*g* for 10 min. The platelet-rich plasma was then harvested by gentle pipetting up to a distance of 4 mm above the buffy coat to avoid contamination with leukocytes. The platelet-rich plasma was then pooled into a 15 ml Falcon tube and centrifuged at 2500×*g* for 20 min. The platelet-poor plasma above the cell pellet was pooled and centrifuged at 3200×*g* for 5 min in a different 15 ml Falcon tube. The platelet-free plasma was again harvested by gently pipetting into a new 15 ml Falcon tube for an additional centrifugation at 3200×*g* for 5 min to ensure maximal removal of platelets. Each time the plasma was transferred into a new Falcon tube, care was taken to avoid touching the cell pellet at the bottom of the tube. Plasma was aliquoted into 100 μL and 500 μL volumes prior to being frozen at − 80 °C.

Patient data recorded at each visit included the erythrocyte sedimentation rate (ESR), C-reactive protein (CRP) levels and simple disease activity index (SDAI)^25^. Rheumatoid factor (RF) and anti-cyclic citrullinated peptide (anti-CCP) levels were also measured at baseline and 12 months. All participants were ≥ 18 years of age. The study was performed according to the Declaration of Helsinki for studies with human subjects. This project was approved by the CHU de Québec-Université Laval research ethics committee (REB # B13-06–1243, REB # 2012–337: 96.05.10 and REB # 2017–2966).

### Fluorescence-activated cell sorting (FACS)

Blood samples were collected in BD vacutainer citrate tubes and buffy coat was prepared as described above. Erythrocytes from an aliquot of buffy coat were lysed with Pharm Lyse™ (BD Transduction Laboratories, Billerica, MA, USA) prior to FACS (BD FACS LSR II sorter) with the antibody cocktail described in Additional file 1. Staining specificity of each fluorochrome was verified with a Fluorescence Minus One (FMO) control and isotypic control antibodies. See Additional file 5 for flow cytometry gating strategy for CLEC12A (MICL for myeloid inhibitory C-type lectin-like receptor).

### Western blot analysis

Neutrophils were purified from blood samples (buffy coat, described above) by negative selection with the EasySep direct neutrophil isolation kit (19,666, STEMCELL Technologies, Vancouver, BC, Canada) according to manufacturer’s recommendations after the removal of platelet-rich plasma. See Additional file 6 and 7 for neutrophil purity (CLEC12A indicated as MICL for myeloid inhibitory C-type lectin-like receptor). Purified neutrophils were resuspended in Hanks' Balanced Salt Solution (HBSS) and lysed prior to SDS-PAGE under non-reducing (for CLEC12A) or reducing (for flotillin-1) conditions as in Gagné et al^16^. Final antibody concentrations for immunoblotting were: 4 µg/ml anti-CLEC12A (clone 50C1, kindly provided by Dr Mireille Lahoud), 0.5 µg/ml anti-flotillin-1 (610821, BD Transduction Laboratories, Canada), and 0.05 µg/ml horseradish peroxidase-labelled donkey anti-mouse (715-035-150, Jackson ImmunoResearch Laboratories Inc., West Grove, PA,USA) antibodies. Staining was detected with the Western Lightning Chemiluminescence Plus ECL kit (PerkinElmer, Waltham, MA, USA) within a maximal exposure time of five minutes.

### Cytokine analysis

Plasma cytokines were quantitated (pg/mL) with the Cytokine and Chemokine 34-Plex Human ProcartaPlex™ Panel 1A’ (EPX340-12167-901, Thermo Fisher, Waltham, MA, USA) as per manufacturer’s instructions.

### Statistical analysis

Descriptive statistics are presented as means with standard deviation or frequency with percentage without missing values for continuous and categorical variables respectively. CLEC12A expression was compared between groups with Student’s *t*-test as well as between visits or between healthy donor and eRA 18 months with generalized estimating equations (GEE) models. When the global test was significant at a 5% level, multiple comparisons were made with Bonferroni correction. Normality and homoscedasticity of the residuals and the absence of outliers were verified in the model. Correlations between CLEC12A expression and clinical parameters were determined with the Spearman’s rank correlation coefficient. Correlations between CLEC12A expression and levels of cytokines detected in the majority of eRA patients or healthy donors were also determined with the Spearman’s rank correlation coefficient. Cytokines not detected in the majority of eRA patients or healthy donors were analyzed as dichotomous variables. The Wilcoxon Mann Whitney test was used to identify correlations between CLEC12A expression and cytokines detected in at least two eRA patients or healthy donors. SAS 9.4 software, version 9.4 (SAS Institute Inc., Cary, NC, USA) was used and graphs generated with GraphPad Prism 7 (GraphPad Software, San Diego, CA, USA).

### Ethics approval and consent to participate

The study was performed according to the Declaration of Helsinki for studies with human subjects. This project was approved by the CHU de Québec-Université Laval research ethics committee (REB # B13-06-1243, REB # 2012-337: 96.05.10 and REB # 2017-2966). Written informed consent was obtained from all the participants.

## Supplementary Information

Supplementary Information.

## Data Availability

The datasets used and/or analyzed during the current study are available from the corresponding author on reasonable request.
