# Supplementary material for: Expression of the myeloid inhibitory receptor CLEC12A correlates with disease activity and cytokines in early rheumatoid arthritis
Source: Sci Rep. 2021 May 27;11:11248. doi: 10.1038/s41598-021-90631-7 (PMC8160002; doi:10.1038/s41598-021-90631-7)
Supplement: Supplementary file 1 — Supplementary Information. [file 41598_2021_90631_MOESM1_ESM.pdf]

SUPPLEMENTARY MATERIAL

Expression of the Myeloid Inhibitory Receptor CLEC12A Correlates with Disease Activity and Cytokines in Early Rheumatoid Arthritis

Myriam Vaillancourt[1,2], Philippe Desaulniers[3], Guillaume Paré[1,2], Nathalie Pagé[1], Asmaa Lachaab[1,2], Anthony Kerever[1,2], Anne-Sophie Julien[4], Nathalie Amiable[1], Martin Pelletier[1,2], Philippe A. Tessier[1,2], Louis Bessette[1,3], Laëtitia Michou[3,5], Paul R. Fortin[1,3] and Maria J. Fernandes[1,2*].

[1] Division of Infectious Diseases and Immunology, CHU de Québec-Université Laval Research Center, Québec City, QC, Canada

[2] Department of Microbiology-Infectious Diseases and Immunology, Faculty of Medicine, Université Laval, Québec City, QC, Canada

[3] Division of Rheumatology, Department of Medicine, CHU de Québec-Université Laval Research Center, Québec City, QC, Canada

[4] Department of mathematics and statistics, Université Laval, Québec City, QC, Canada

[5] Division of Endocrinology et Nephrology, CHU de Québec-Université Laval Research Center, Québec City, QC, Canada

*Corresponding author: Maria J. Fernandes, Centre de recherche du CHU de Québec-Université Laval, 2705 boulevard Laurier, Room T1-49, Québec, QC, Canada, G1V 4G2, maria.fernandes@crchudequebec.ulaval.ca

ORCID (MP): 0000-0001-5033-4947

SUPPLEMENTARY MATERIAL

Additional file 1: Antibodies used for staining blood cells for flow cytometry analysis

Antibody	Fluorochrome	Clone	Company	Cat. Number	Target cells
CD16	PerCP-eFluor 710	3G8	Thermo Fisher	46-0166	Neutrophils
CD36	FITC	eBioNL07	Thermo Fisher	11-0369	Monocytes
CD193 (CCR3)	PE	eBio5E8-G9-B4	Thermo Fisher	12-1939	Eosinophils
CD56 (NCAM)	PE/Cyanine5	CMSSB	Thermo Fisher	15-0567	NK cells
CD66b	PE/Cyanine7	G10F5	Thermo Fisher	25-0666	Granulocytes
CD371 (CLEC12A)	APC	50C1	BioLegend	353605	Myeloid cells
CD64 (FcγR1)	APC/eFluor 780	10.1	Thermo Fisher	47-0649	Monocytes & activated neutrophils
Fixable Viability Dye	eFluor 455UV	N/A	Thermo Fisher	65-0868	N/A
CD3	eVolve 655	OKT3	Thermo Fisher	86-0037	T cells
CD45	eFluor450	2D1	Thermo Fisher	48-9459	Leukocytes
CD19	eVolve 605	SJ25C1	Thermo Fisher	83-0198	B cells

SUPPLEMENTARY MATERIAL

Additional file 2: Cytokine Analysis of the early RA and healthy donor cohort

**Plasma cytokine**	**Donor type**	**n**	**Mean** (pg/mL)	**SD**
IL-18	eRA - baseline	15	14.15	13.61
	Healthy	10	15.87	11.44
IL-1 RA	eRA - baseline	15	754.45	905.89
	Healthy	10	136.08	150.63
IL-1β	eRA - baseline	15	1.92	2.22
	Healthy	10	0.25	0.80
Eotaxin	eRA - baseline	15	31.72	14.27
	Healthy	10	27.69	10.23
IP-10	eRA - baseline	15	59.34	142.96
	Healthy	10	11.26	5.01
MCP-1	eRA - baseline	15	30.93	11.29
	Healthy	10	20.19	6.58
MIP-1α	eRA - baseline	15	13.80	18.49
	Healthy	10	16.10	17.03
MIP-1β	eRA - baseline	15	135.54	156.00
	Healthy	10	63.99	119.32
RANTES	eRA - baseline	15	81.49	54.55
	Healthy	10	37.91	49.66
SDF-1α	eRA - baseline	15	2152.18	4082.27
	Healthy	10	451.00	192.31

eRA early arthritis; *n* sample number

Additional file 3: Comparison of neutrophil and monocyte CLEC12A expression of healthy donors and eRA patients at 18 months

	**Neutrophil CLEC12A**		**Monocyte CLEC12A**	
	Expression	SD	Expression	SD
Healthy donors	2774.31	475.80	4769.69	667.84
eRA (18 months)	2209.18	318.83	5103.18	432.56

P value HD vs eRA = 1.00 (NS)

SUPPLEMENTARY MATERIAL

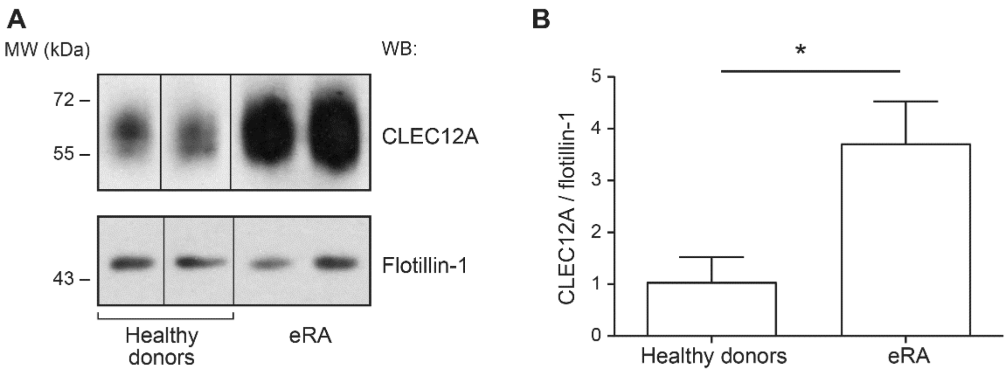

Additional file 4: CLEC12A expression in eRA patients at baseline and healthy donor neutrophils. Peripheral blood neutrophils were purified and lysed before immunoblotting with the 50C1 anti-CLEC12A antibody. ***(A)*** A representative blot of neutrophils of two different healthy donors *(first two lanes)* and two eRA patients with the highest level of CLEC12A expression at baseline as determined by flow cytometry *(last two lanes)* is shown. Flotillin-1 expression was used as a loading control. All CLEC12A samples were migrated on the same gel, under non-reducing conditions (see the original blot on the next page). The lines delineate the lanes of the gel that were cropped to group the healthy donor samples next to the patient samples. ***(B)*** Densitometry analysis of neutrophil CLEC12A expression of the 4 patients with the highest level of expression of CLEC12A (lanes 5, 6, 7, 9) as determined by flow cytometry compared to 4 healthy donors (lanes 1-4). Statistical analysis: Student's t-test (p = 0.025)

SUPPLEMENTARY MATERIAL

Original blot of figure in Additional file 4:

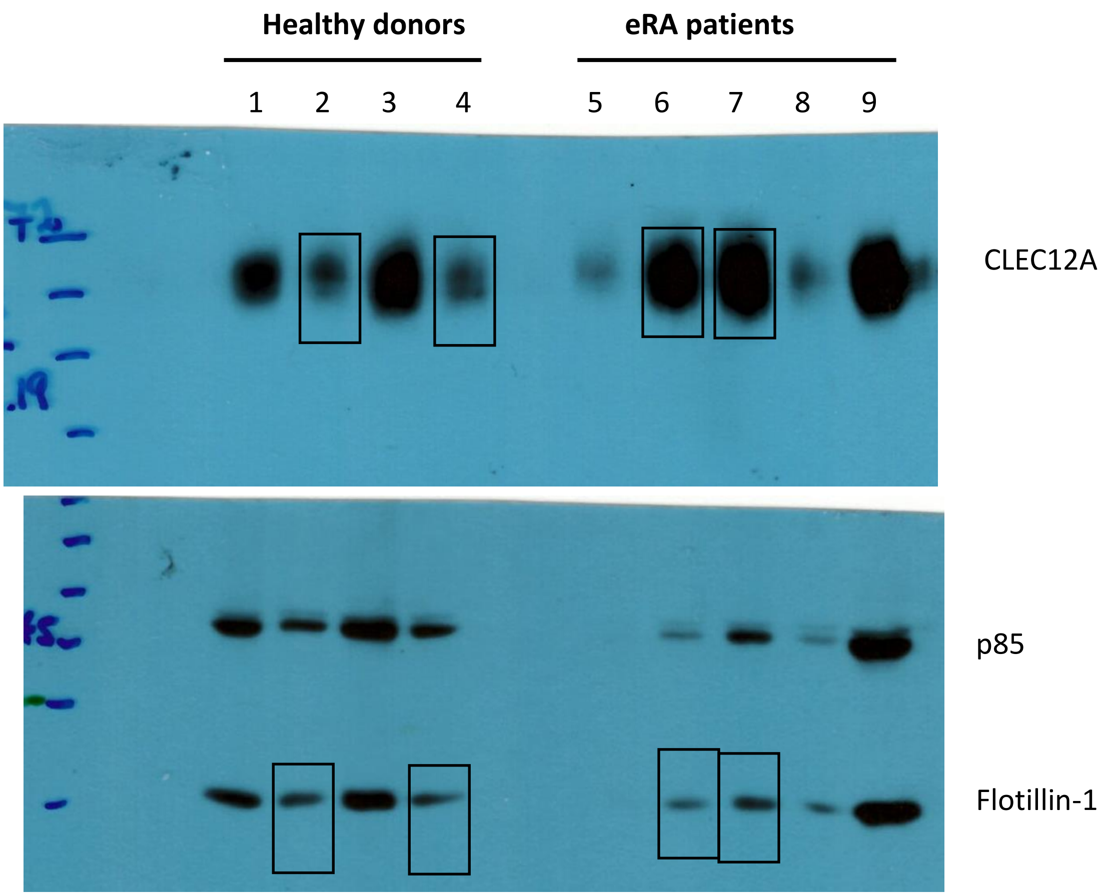

Note: The sample in lane 8 corresponds to the neutrophil lysate of an eRA patient whose level of CLE12A expression is below the mean of CLEC12A expression of healthy donors. The intensity of the CLEC12A band in lane 5 is low since we had very few neutrophils for this patient in our lysate and thus much less material in the well to load as seen by the loading controls flotillin-1 and p85. This patient expresses higher levels of CLEC12A than healthy donors by flow cytometry.

Regarding the blotting of the membranes, all samples were migrated on the same gel, under non-reducing conditions and transferred to a PDVF membrane. Blotting was performed on the full length membrane with each antibody. The membrane was not cut prior to hybridization. The lines delineate the lanes of the gel that were cropped to group the healthy donor samples next to the patient samples in the figure in Additional file 4.

SUPPLEMENTARY MATERIAL

We show here the other film we have for this experiment in our files. It was exposed for a shorter time.

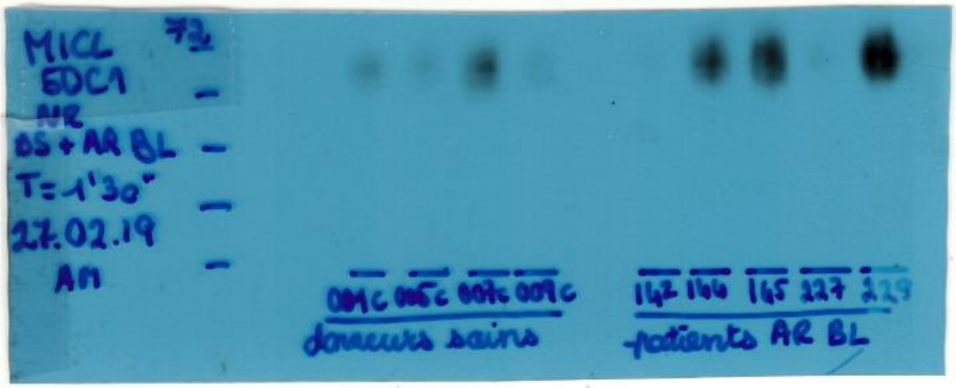

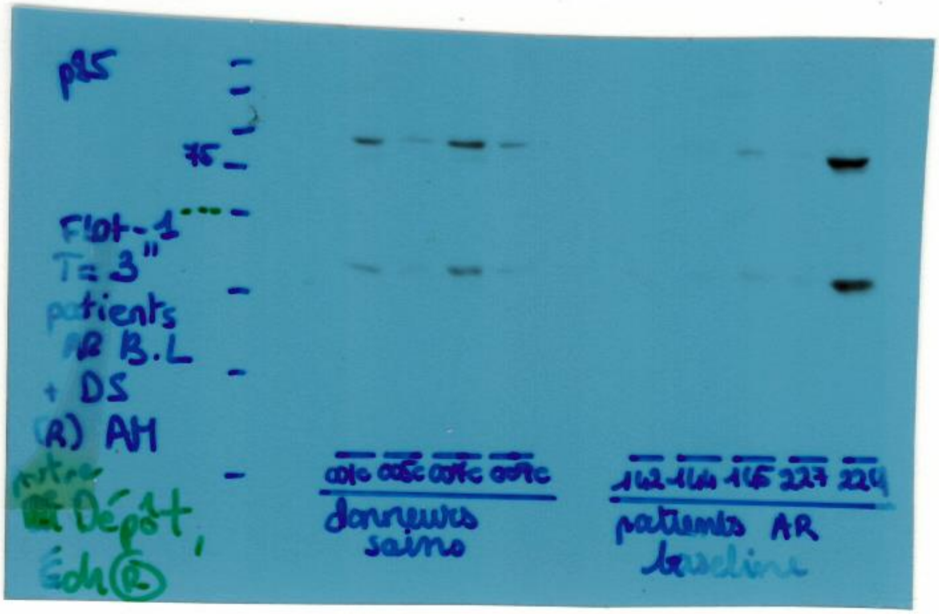

SUPPLEMENTARY MATERIAL

Additional file 5: Flow cytometry gating strategy

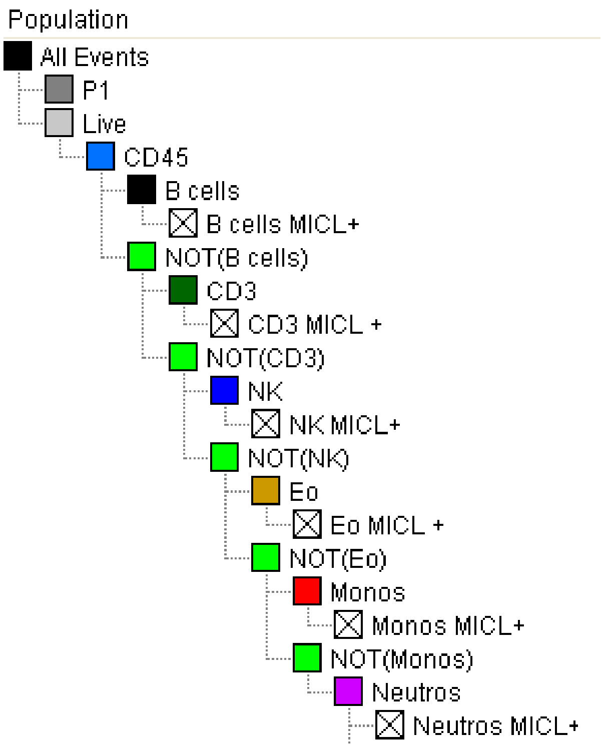

Legend:

Leukocytes = $CD45^+$

B cells = $CD19^+$

T cells = $CD3^+$

NK cells = $CD56^+$ (NCAM)

Eosinophils (Eo) = $CD193^+$ (CCR3)

Monocytes (Monos) = $CD36^+$

Neutrophils (Neutros) = $CD16b^+$

SUPPLEMENTARY MATERIAL

Additional file 6: Purity of healthy donor neutrophils isolated with the Stem Cell EasySep direct neutrophil isolation kit

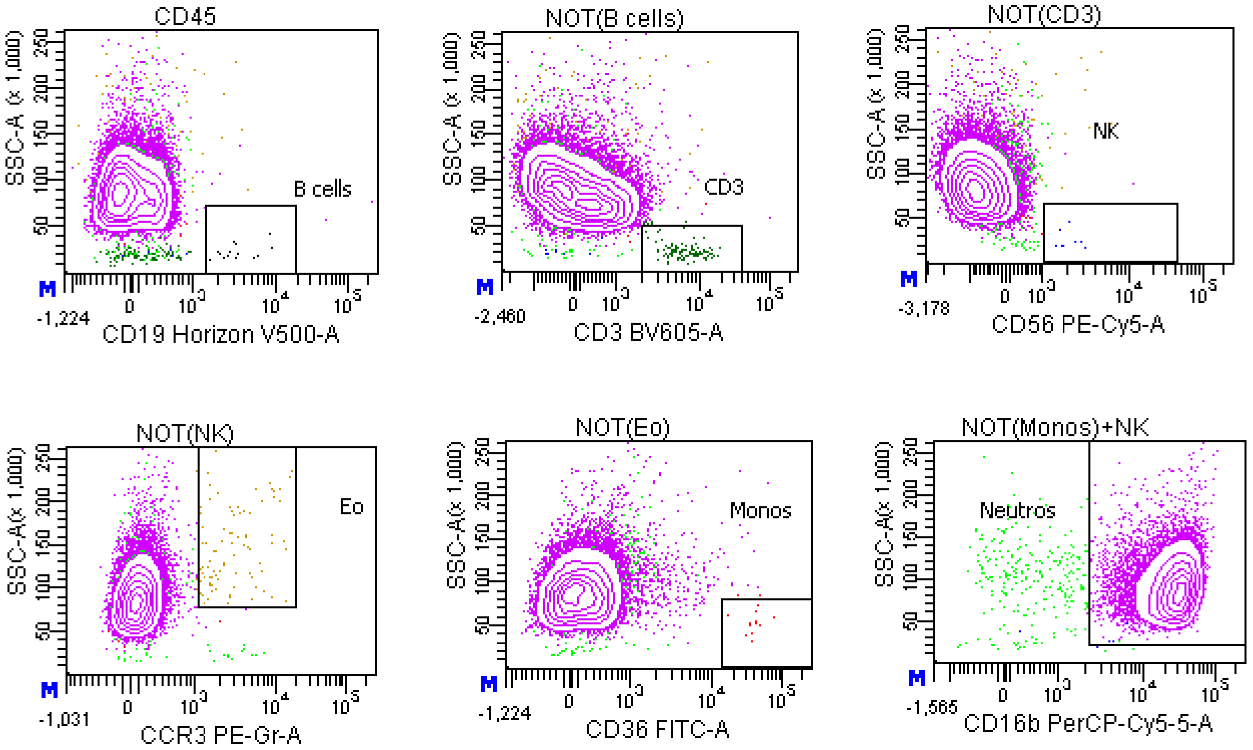

Population	#Events	%Parent	%Total
All Events	15,203	####	100.0
P1	55	0.4	0.4
Live	15,130	99.5	99.5
CD45	15,130	100.0	99.5
B cells	14	0.1	0.1
B cells MICL+	0	0.0	0.0
NOT(B cells)	15,116	99.9	99.4
CD3	117	0.8	0.8
CD3 MICL +	3	2.6	0.0
NOT(CD3)	14,999	99.2	98.7
NK	10	0.1	0.1
NK MICL+	2	20.0	0.0
NOT(NK)	14,989	99.9	98.6
Eo	105	0.7	0.7
Eo MICL +	78	74.3	0.5
NOT(Eo)	14,884	99.3	97.9
Monos	14	0.1	0.1
Monos MICL+	13	92.9	0.1
NOT(Monos)	14,870	99.9	97.8
Neutros	14,613	98.3	96.1

SUPPLEMENTARY MATERIAL

Additional file 7: Purity of eRA neutrophils isolated with the Stem Cell EasySep direct neutrophil isolation kit

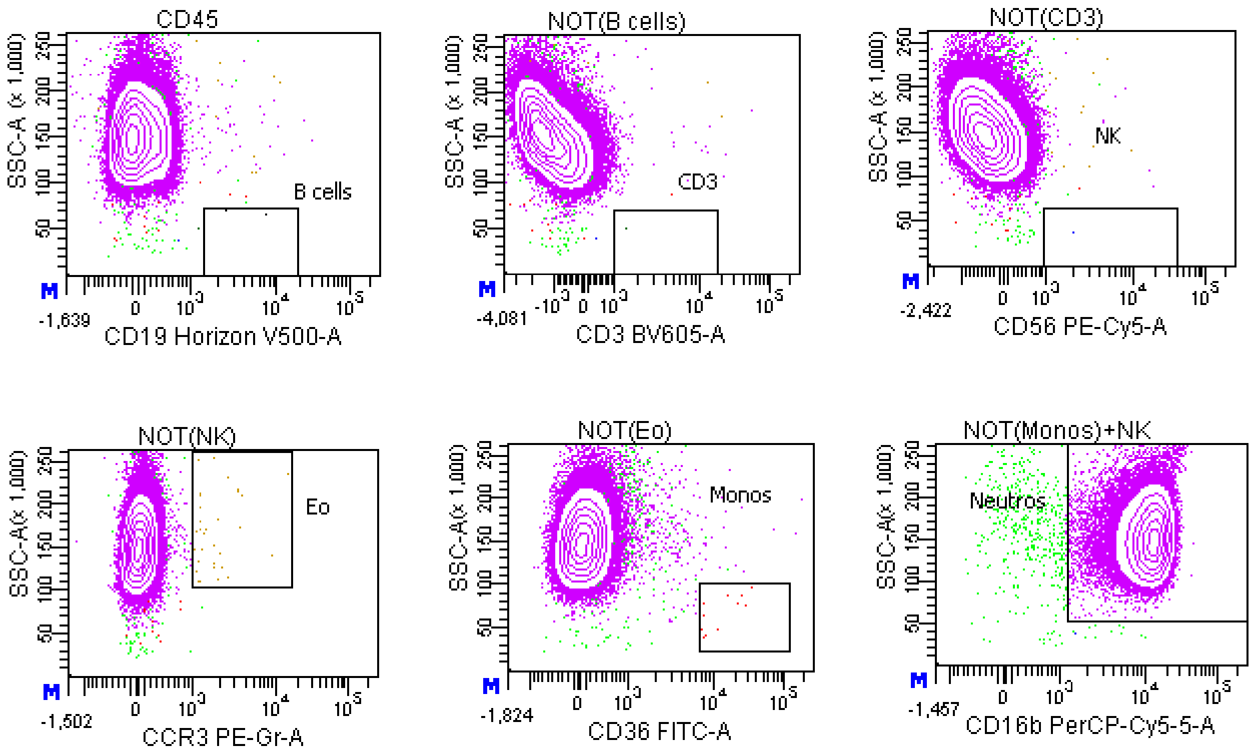

Population	#Events	%Parent	%Total
All Events	50,189	####	100.0
P1	100	0.2	0.2
Live	50,056	99.7	99.7
CD45	50,035	100.0	99.7
B cells	2	0.0	0.0
B cells MICL+	1	50.0	0.0
NOT(B cells)	50,033	100.0	99.7
CD3	2	0.0	0.0
CD3 MICL +	2	100.0	0.0
NOT(CD3)	50,031	100.0	99.7
NK	1	0.0	0.0
NK MICL+	1	100.0	0.0
NOT(NK)	50,030	100.0	99.7
Eo	32	0.1	0.1
Eo MICL +	32	100.0	0.1
NOT(Eo)	49,998	99.9	99.6
Monos	14	0.0	0.0
Monos MICL+	12	85.7	0.0
NOT(Monos)	49,984	100.0	99.6
Neutros	49,536	99.1	98.7
